# Effects of mulching film on soil microbial diversity and community of cotton

**DOI:** 10.1186/s13568-022-01374-1

**Published:** 2022-03-11

**Authors:** Qiuxiang Tang, Tao Lin, Zhanbin Sun, An Yan, Jusong Zhang, Pingan Jiang, Fengquan Wu, Hao Zhang

**Affiliations:** 1 College of Agronomy, Engineering Research Centre of Cotton, Xinjiang Key Laboratory of Grassland Restoration and Environmental Information, Ministry of Education, Xinjiang Agricultural University, Xinjiang, 830052 China; 2 Institute of Cash Crops, Key Laboratory of Crop Ecophysiology and Cultivation in Desert Oasis, Ministry of Agriculture and Rural Affairs, Xinjiang Academy of Agricultural Sciences, Xinjiang, 830091 China; 3 Institute of Environment and Sustainable Development in Agriculture, Key Laboratory of Dryland Agriculture, Ministry of Agriculture and Rural Affairs, Chinese Academy of Agricultural Sciences, Beijing, 100081 China; 4 School of Light Industry, Beijing Technology and Business University, Beijing, 100048 China

**Keywords:** Cotton, PE mulching film, PBAT mulching film, Soil microbial diversity and community

## Abstract

Different types of mulching film could variously influence soil properties and plant growth. Yet, surprisingly few studies have investigated the effects of mulching film upon soil microbial diversity and community structure. In this research, two kinds of mulching film, a traditional PE (polyethylene) mulching film and a degradable PBAT ((Poly [butyleneadipate-*co*-terephthalate])) mulching film, were applied to cotton (*Gossypium* spp.) plants grown in Xinjiang Province, China. The respective influence of the two mulching films on the cotton’s soil microbial (bacteria and fungi) diversity and community were investigated. The results showed that applying the PBAT mulching film could significantly alter the diversity of non-rhizosphere soil fungi when compared to using the PE mulching film. However, neither the PE nor PBAT mulching film had any significant influence on the diversity of soil bacteria and rhizosphere soil fungi. Nevertheless, soil microbial community composition differed under the PBAT mulching film treatment vis-à-vis the PE mulching film treatment. The abundance of *Gibellulopsis* was higher under the PBAT than PE mulching film treatment. Our study’s findings provided an empirical basis for the further application of degradable PBAT mulching film for the sustainable development of cotton crops.

## Key points

- Degradable mulching film alter the diversity of cotton non-rhizosphere soil fungi.
- Degradable mulching film alter the soil microbial composition of cotton.
- Degradable mulching film dose not alter the diversity of cotton rhizosphere fungi.
- Degradable mulching film dose not alter the diversity of cotton soil bacteria.

## Introduction

Cotton (*Gossypium* spp.) is one of the world’s most important crops, having wide applications in agriculture, textile industry, and pharmaceutical industry, to name a few areas (Feng et al. 2017; Song et al. 2020). As the largest cotton production region, Xinjiang Province’s cotton planting area and yield accounts for 78.9% and 87.3% of China’s totals, respectively (China statistical yearbook 2020). The unique geographical location and natural conditions of Xinjiang Province are very suitable for growing cotton. Usage of plastic mulching in cotton planting is now an essential cultivation technique in Xinjiang Province (Li et al. 2018). The application of mulching offers multiple advantages, namely increasing the water application efficiency and ground temperature, and reducing the incidence of cotton diseases, thereby improving the quantity and quality of cotton yield in Xinjiang Province (Dong et al. 2019; Ma et al. 2021).

The most commonly used mulch for cotton’s growth is made of PE (polyethylene) material. This PE mulching film has been shown to play important roles in cotton plants’ growth and development. However, because natural degradation of the PE material in soil is difficult, most of it stays in soil once plowed; hence, long-term usage of PE mulching films cause serious environmental pollution. Moreover, PE mulching film can adversely affect both soil structure and the microbial community composition in soil. In cotton farmland soil, the microplastics from PE mulching film significantly altered its bacterial community (Zhang et al. 2019). Alternatively, using a mulching film that degrades more easily has the advantage of being environmental friendly, and its usage in cotton production is gaining popularity. Degradable mulching film had benefit to cotton yield and soil properties. Application of degradable mulching film could significantly improve the cotton yield compared with non-mulched treatment, but had no notably difference with plastic mulch film (Deng, et al. 2019; Wang et al. 2019). Moreover, soil water content and salinity in degradable mulching film application was higher than usage of plastic mulch film in certain period (Wang et al. 2019).

Soil microbial characteristics could reflect the healthy status of soil, which is vital for the sustainable development of cotton farming (Wang et al. 2021). Many studies have demonstrated that the usage of plastic mulching is capable of influencing the diversity and community composition of soil microbiota. Recently, Dong et al. (2017) found that mulching applications could significantly increase soil microbial diversity and alter soil microbial community composition in a rain-fed region of northeastern China. A similar phenomenon was reported from a semi-arid orchard system, where the mulching application considerably augmented soil fungi diversity and abundance, and positively affected soil bacterial richness in an apple tree orchard (Wang et al. 2020). Moreover, the type of mulching film used can differentially impact soil microbial community composition and diversity. For example, Zhang et al. (2020) found that, compared with polyethylene film mulching application, peanut hull mulching in a tea plantation could significantly improve the abundance of the phylum Nitrospirae in its soil bacterial communities, and that of Mortierellomycota and Basidiomycota in soil fungal communities. However, seldom do studies assess how different mulching films affect soil microbial community and diversity across multiple stages of cotton plants’ growth.

To investigate the influence of different kinds of mulching film upon soil microbial community and diversity, two types, the traditional PE mulching film and a degradable PBAT (Poly [butyleneadipate-co-terephthalate]) mulching film were used in this study. The main difference between the two mulching films is the composition. PE mulching film was composed of polyethylene, which is difficult to degrade. PBAT mulching film was composed of poly butyleneadipate-co-terephthalate, which could be degraded. Meanwhile, the influence of these two mulching films on microbial diversity and community composition of cotton’s rhizosphere soil and non-rhizosphere soil at boll opening stage was evaluated. This research provides evidence favoring the further application of degradable mulching film when growing cotton.

## Methods and materials

### Experimental site and design

The field experiment was conducted at the Xinjiang Academy of Agricultural Sciences’ cotton comprehensive experiment site, located in Xinjiang Province (40°06′ N, 80°44′ E). This experimental site has a typical temperate continental arid climate, with evaporation amount, sunshine duration, average annual precipitation and temperature of 2900 mm, 2679 h, 46.7 mm and 10.4 ℃, respectively.

The main testing plot is 14-m long and 6.84-m wide, whose soil is silt loam. The average dry bulk density is 1.48 g/cm^3^, the content of organic matter and total nitrogen in soil is 10.6 g/kg and 1.79 g/kg, respectively. Two types of mulching film, PE and degradable PBAT, were used. Cotton planting was managed via mechanical laminating and sowing. Each treatment was repeated three times.

### Soil sampling

The two treatments were designated as follows: PE mulching film application at the boll opening stage (PEBOS), and the degradable PBAT mulching film application at the boll opening stage (DPBOS).

Two soil types, rhizosphere soil and non-rhizosphere soil, were collected from the treatments. Cotton roots were taken out, and then a hairbrush was used to gently dislodging the soil adhering to cotton roots to collect rhizosphere soil. From every treatment replicate plot, soil was collected from 0 to 10 cm depth at seven points randomly, then mixed and passed through sieve to form composite samples of the non-rhizosphere soil. Both the two type soils were preserved and used for soil microbial diversity and community analysis.

### Soil DNA extraction and PCR amplification

From each sample, soil DNA was extracted with a FastDNA Spin Kit for soil (MP, Santa Ana, USA), according to the manufacturer’s instructions. The purity and concentration of soil DNA was detected on a NanoDrop2000 (Thermo Scientific, Wilmington, USA). The integrity of soil DNA was checked with 1% agarose gel.

The primer pair of 338F (5′-ACTCCTACGGGAGGCAGCAG-3′) and 806R (5′-GGACTACHVGGGTWTCTAAT-3′) was used to amplify the V3–V4 region of 16S rDNA (Xu et al. 2016). A 20-μL PCR reaction system contained 4 μL of 5 × FastPfu Buffer, 2 μL of 2.5 mM dNTPs, 0.8 μL each of the 338F and 806R primers, 0.4 μL of FastPfu polymerase, 10 ng of template DNA, 0.2 μL of BSA, and residual ddH_2_O. The PCR reaction program went as follows: 95 °C for 3 min, then 27 cycles at 95 °C for 30 s, 55 °C for 30 s, and 72 °C for 45 s, with a final extension at 72 °C for 10 min.

The primer pair of ITS1F (5′-CTTGGTCATTTAGAGGAAGTAA-3′) and ITS2R (5′-GCTGCGTTCTTCATCGATGC-3′) was used to amplify the ITS1 region of ITS rDNA (Adams et al. 2013). The 20-μL PCR reaction system contained 2 μL of 10 × Buffer, 2 μL of 2.5 mM dNTPs, 0.8 μL each of the ITS1F and ITS2R primers, 0.2 μL of rTaq polymerase, 10 ng of template DNA, 0.2 μL of BSA, and residual ddH_2_O. The conditions of the PCR reaction program were as follows: 95 °C for 3 min, with 35 cycles at 95 °C for 30 s, 55 °C for 30 s, and 72 °C for 45 s, ending with an extension at 72 °C for 10 min.

### Illumina Mi-Seq sequencing

The Mi-Seq libraries were derived by a TruSeqTM DNA Sample Prep Kit (Illumina, San Diego, CA, USA) and following the manufacturer’s instructions. All the constructed libraries were then sequenced on the Illumina Mi-Seq platform, this done by the Majorbio Co. Ltd. (Shanghai, Chia). The ensuing raw sequence data were filtered as previously described (Sun et al. 2021). All the sequences data were uploaded in NCBI Sequence Read Archive database.

### Bioinformatics analysis

Several well-known indexes were derived to assess differences in microbial diversity and community composition between the two kinds of mulching film tested in the cotton field. The Sobs, Shannon, Simpson, Ace, and Chao indexes were used to express alpha diversity. Student’s t-test was used to analyze the alpha diversity. Principal coordinate analysis (PCoA) was used to investigate the microbial beta diversity among all samples (Wen et al. 2020).

## Results

### Mi-Seq sequencing analysis

For fungi, a total of 415,816 sequences were obtained from the rhizosphere soil samples after undergoing Illumina Mi-Seq sequencing for the ITS1 region of ITS rDNA. Their average length was 228 bp. From the non-rhizosphere soil samples, a total of 408,590 sequences were generated, having an average length of 230 bp.

For bacteria, 426,303 sequences in all were obtained from the non-rhizosphere soil samples after their Illumina Mi-Seq sequencing of the 16S rDNA V3–V4 region; these had an average length of 416 bp. From the rhizosphere soil samples, 363,833 sequences were generated overall, with an average length of 416 bp.

### Microbial community composition

Two taxonomic strata, phylum and genus, were used to examine similarities and differences in the microbial communities under the two mulching film treatments.

**Fig. 1:**
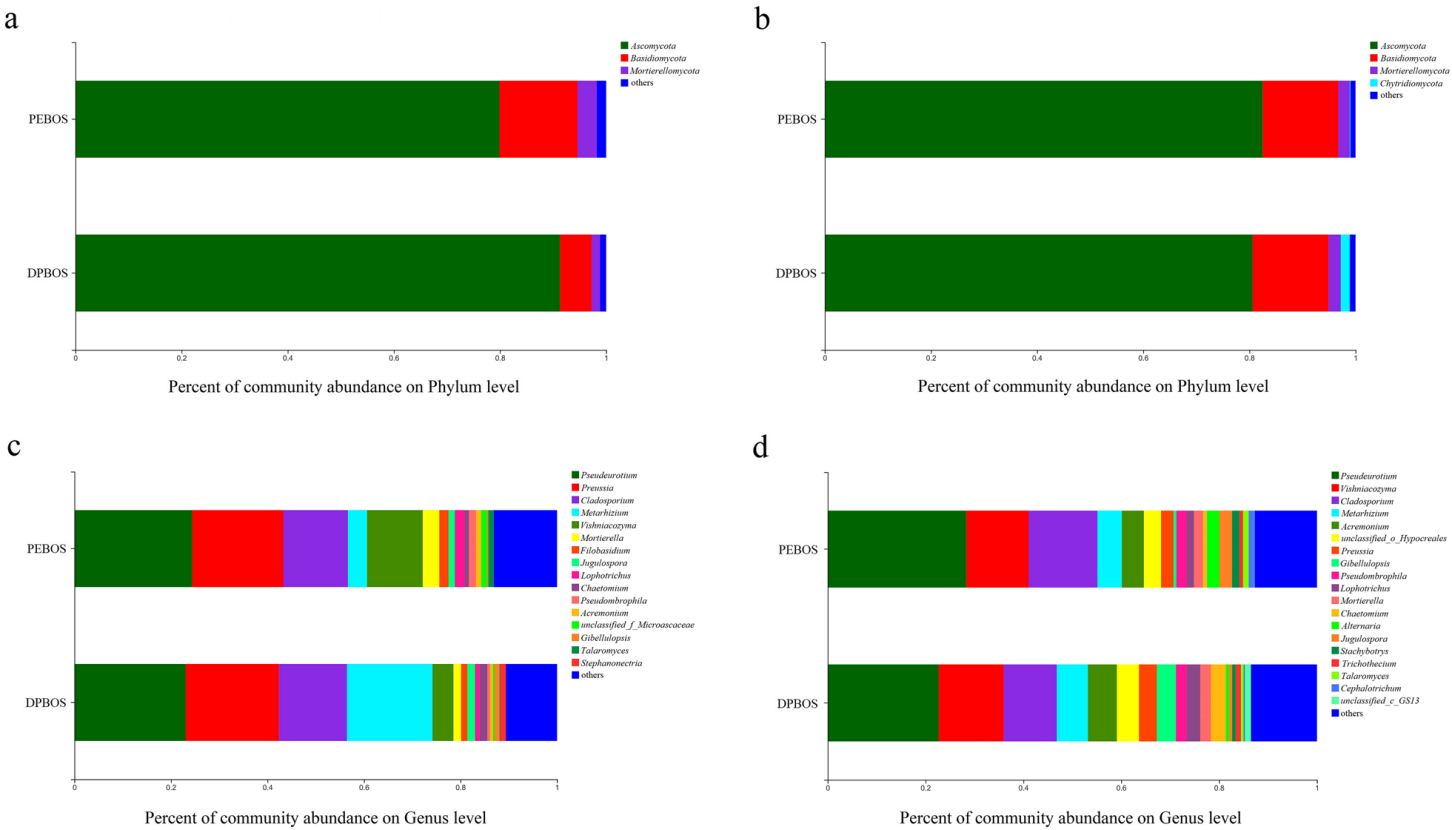
Soil fungal community composition under the application of two different mulching films. On the x-axis is the percentage of community abundance, and on the y-axis the treatments. **a** Rhizosphere soil fungal community at the phylum level, **b** non-rhizosphere soil fungal community at the phylum level, **c** rhizosphere soil fungal community at the genus level, and **d** non-rhizosphere soil fungal community at the genus level

Both treatments exhibited a similar dominant fungal community at the level of phylum dwelling in the rhizosphere and non-rhizosphere soils. The *Ascomycota*, *Basidiomycota*, and *Mortierellomycota* were the most dominant phyla. However, differences emerged at the genus level in the rhizosphere and non-rhizosphere soil between the two treatments (Fig. 1a, b).

The dominant fungal genera in the rhizosphere soil of PEBOS were *Pseudeurotium* (24.4%), *Preussia* (18.9%), and *Cladosporium* (13.4%), whereas *Metarhizium* (17.7%) in rhizosphere soil was higher in the DPBOS than PEBOS treatment (4.0%). The dominant genera present in the non-rhizosphere soil of DPBOS were *Pseudeurotium* (22.7%), *Vishniacozyma* (13.3%), and *Cladosporium* (10.8%). Under the PEBOS treatment, the most dominant genus was *Pseudeurotium* (28.3%), followed by *Cladosporium* (14.1%) and *Vishniacozyma* (12.7%) (Fig. 1c, d).

**Fig. 2:**
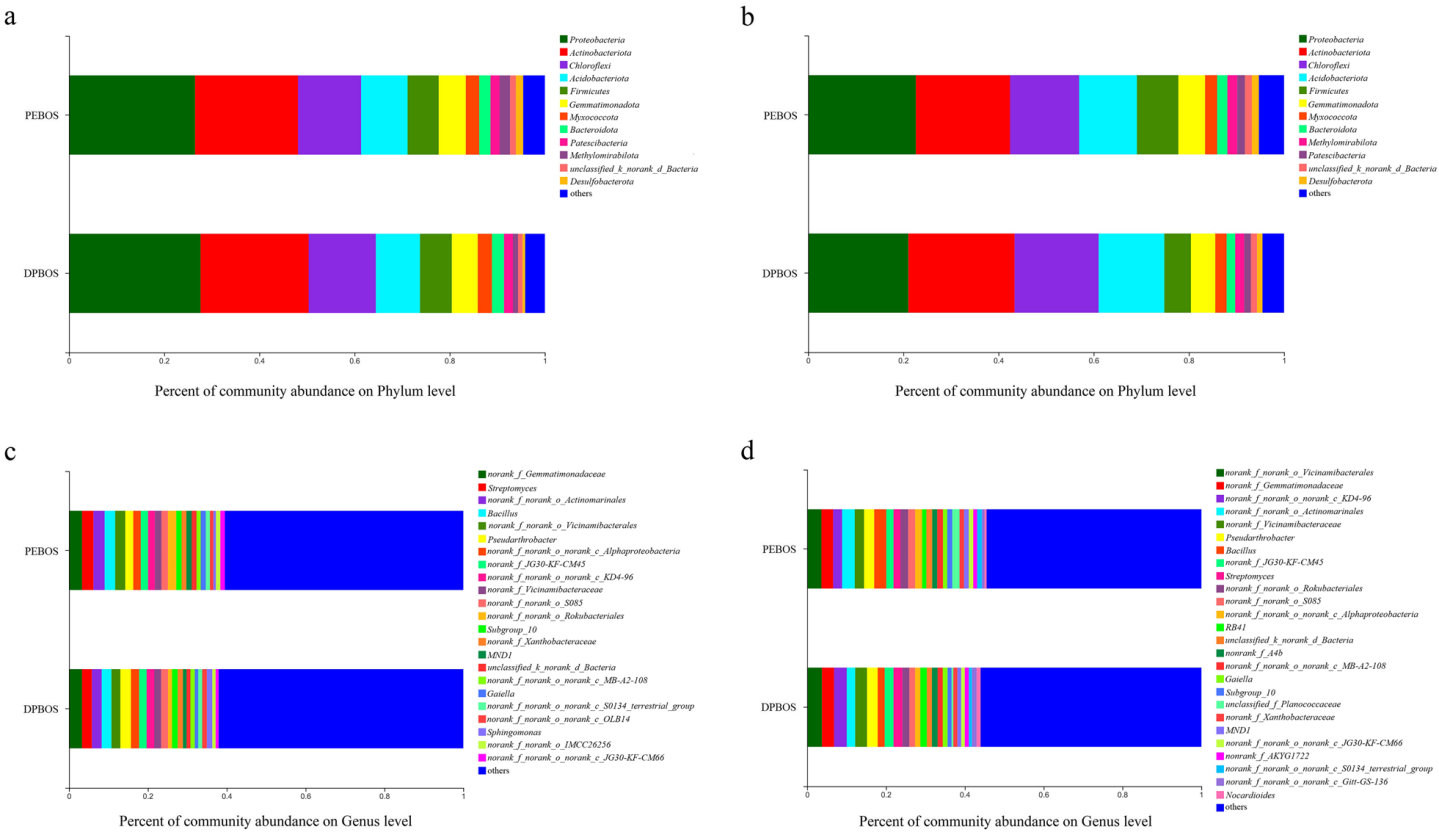
Soil bacterial community composition under the application of two different mulching films. On the x-axis is the percentage of community abundance, and on the y-axis the treatments. **a** Rhizosphere soil bacterial community at the phylum level, **b** non-rhizosphere soil bacterial community at the phylum level, **c** rhizosphere soil bacterial community at the genus level, and **d** non-rhizosphere soil bacterial community at the genus level

The *Proteobacteria*, *Actinobacteriota*, *Chloroflexi*, and *Acidobacteriota* were the most dominant bacterial phyla between the two treatments in the rhizosphere and non-rhizosphere soil samples (Fig. 2a, b).

The most abundance bacterial genera in the rhizosphere soil of PEBOS were *norank_f_Gemmatimonadaceae* (3.3%). Similarly, the most abundant bacterial genera in the DPBOS treatment were same to the PEBOS treatment. In stark contrast, evidently the non-rhizosphere soil harbored distinctive dominant taxa (Fig. 2c, d). The most abundance bacterial genera in the non-rhizosphere soil of PEBOS were *norank_f_norank_o_Vicinamibacterales* (3.6%), and those of the DPBOS treatment were also *norank_f_norank_o_Vicinamibacterales* (3.8%). The abundance of *Bacillus* was higher in PEBOS than DPBOS in both rhizosphere and non-rhizosphere soil. For *Streptomyces*, the abundance in DPBOS is higher than PEBOS in non-rhizosphere soil.

### Microbial α-diversity

**Fig. 3:**
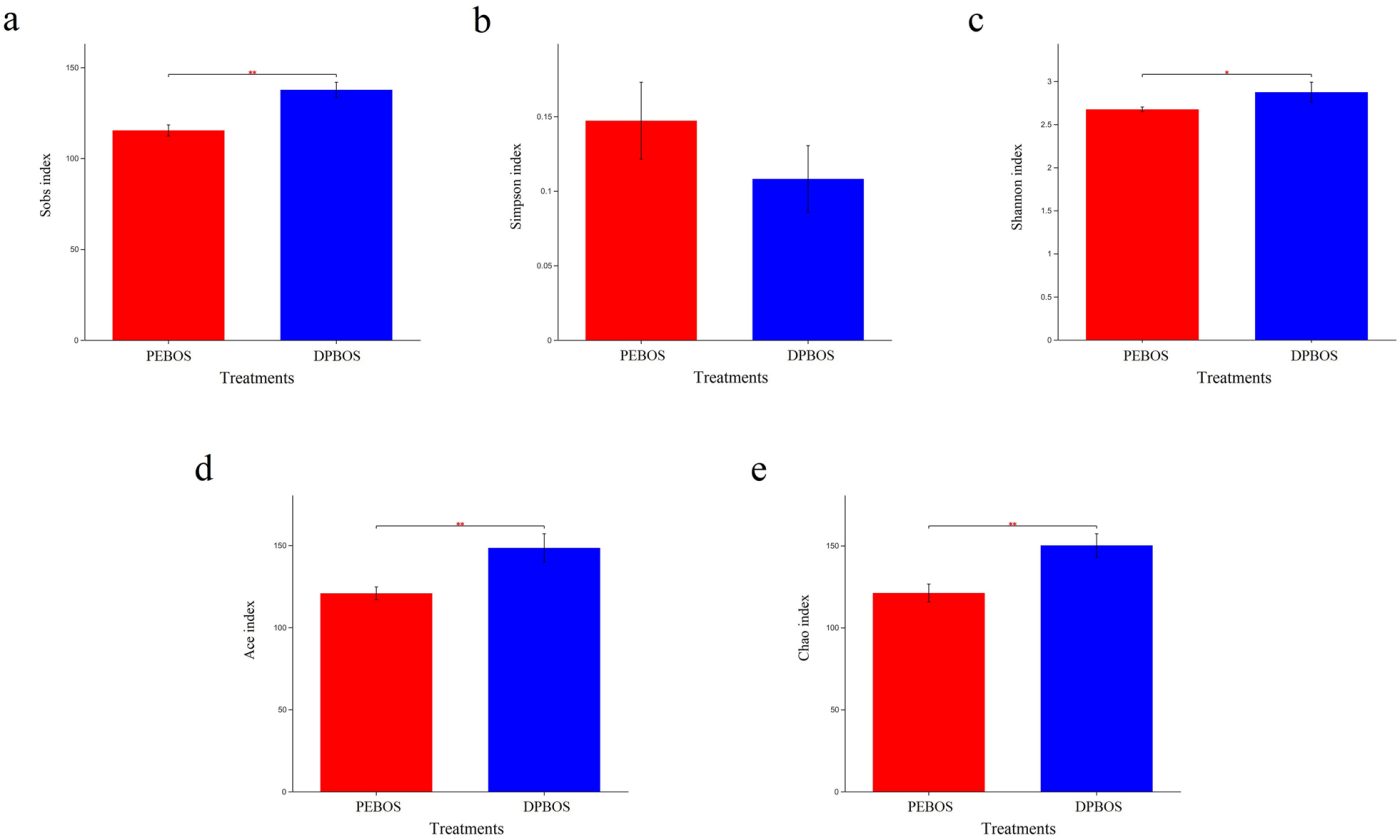
Alpha diversity analysis of non-rhizosphere soil fungal communities under the application of two different mulching films. The x- and y-axis represent the treatments and diversity indexes, respectively. *p < 0.05, **p < 0.01. **a** Sobs index. **b** Simpson index. **c** Shannon index. **d** Ace index, and **e** Chao index

Five indexes—Sobs, Shannon, Simpson, Ace, and Chao were used to convey the diversity and richness under each of the two mulching treatments. For non-rhizosphere soil, the Sobs, Shannon, Ace, and Chao values for the fungal community differed significantly between the DPBOS and PEBOS treatments, indicated that the diversity and richness of non-rhizosphere soil fungal was altered considerably by the type of mulching film applied to grow cotton plants (Fig. 3). For rhizosphere soil’s fungal community, no significant differences were discernible between the DPBOS and PEBOS treatments.

Moreover, no significant difference was detected in the bacterial community between DPBOS and PEBOS treatments in either the rhizosphere and non-rhizosphere soils. This indicated that application of the PE mulching film negligibly influences the bacterial diversity and richness when compared with using degradable or PBAT mulching film, either in rhizosphere or non-rhizosphere soils.

### Microbial β-diversity

**Fig. 4:**
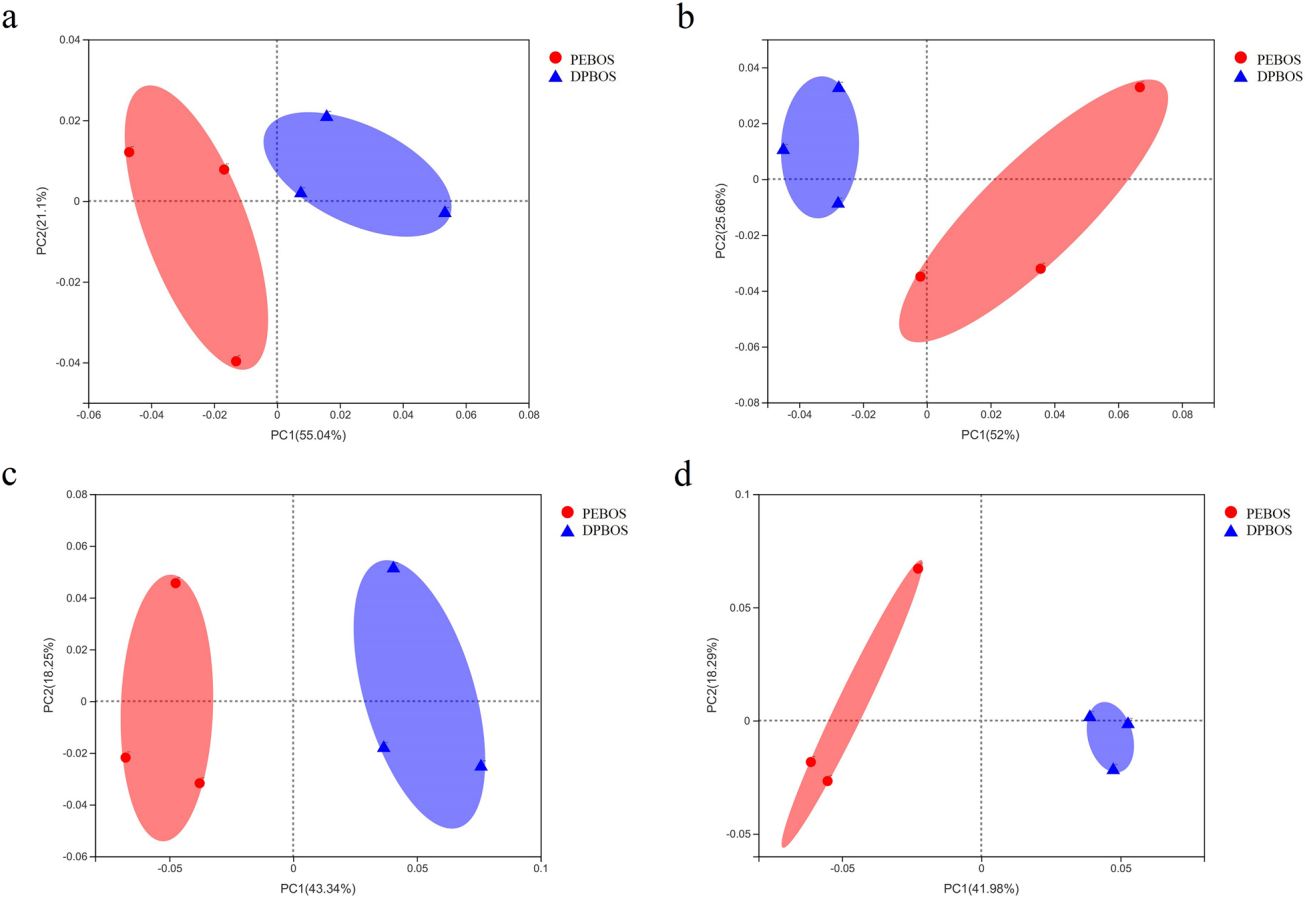
Principal coordinates analysis (PCoA) of soil fungal communities under the application of two different mulching films. **a** Rhizosphere soil fungal community; **b** non-rhizosphere soil fungal community. **c** Rhizosphere soil bacterial community; **d** non-rhizosphere soil bacterial community. The x-axis and y-axis represent the variance in soil fungal structures. The PEBOS and DPBOS, respectively denote the PE mulching film and degradable PBAT mulching film treatment

PCoA was used to infer divergence in microbial community structure between treatments. According to the PCoA results, the bacterial community under the DPBOS treatment was separated from PEBOS treatment, in both the rhizosphere and non-rhizosphere soil zones, which indicated that bacterial community structure was significantly altered by the type of mulching film used (Fig. 4a, b). The fungal community showed a similar response to the DPBOS treatment versus PEBOS treatment as the bacterial community. That is, the fungal community structure in DPBOS treatment was likewise separated from PEBOS treatment in both rhizosphere and non-rhizosphere soil, suggesting that fungal community structure was remarkably affected by the application of different mulching films (Fig. 4c, d).

## Discussion

The kind of mulching film used could influence the diversity and community of soil microbes, which are important determinants of soil properties and plants’ growth. Therefore, a better understanding of how mulching film changes soil microbial diversity and community is crucial for the sustainable development of agriculture. In our study, two mulching film types, PE and degradable PBAT materials, were used to investigate the belowground responses in terms of the diversity and community of rhizosphere and non-rhizosphere soil microbes in a cotton-growing field. We found that the diversity of rhizosphere and non-rhizosphere soil bacteria did not depend on whether PE or a degradable PBAT mulching film was used, whereas the diversity and richness of non-rhizosphere soil fungi clearly did, being significantly different PEBOS and DPBOS treatment. A plausible explanation for the latter is the variety of soil fungi that associated with the ability to degrade PBAT materials in DPBOS was quit different with PEBOS. Different mulching film materials could also influence the diversity of soil fungi. Similar phenomenon had been found in tea plantation under different mulching patterns. Compared with polyethylene film mulching, application of peanut hull mulching increased the diversity of soil fungal community (Zhang et al. 2020).

Both rhizosphere and non-rhizosphere soil fungal communities were altered by applying the degradable PBAT mulching film in comparison with the PE mulching film. The genus *Gibellulopsis* was more abundant in the DPBOS treatment than in the PEBOS treatment in both rhizosphere and non-rhizosphere soil. *Gibellulopsis* had been previously isolated from compost, rotting root soil, and decomposing grains (López et al. 2021; Kotova et al. 2016; Tian et al. 2021). *Gibellulopsis* is reportedly associated with carbohydrate content. Therefore, we speculate that *Gibellulopsis* in DPBOS treatment soil was related to PBAT’s decomposition, and that its presence could accelerate the degradation of PBAT mulching film.

Unlike soil fungal community composition, soil bacterial community composition was only slightly altered after the application of degradable PBAT mulching film and PE mulching film. In particular, the abundance of *Streptomyces* was higher in the DPBOS than PEBOS treatment in non-rhizosphere soil. *Streptomyces* is able to degrade cellulose, pesticides, and petroleum compounds (Chen et al. 2020; Ohashi et al. 2021; Santillan et al. 2020). Further, *Streptomyces* may be critically involved in plastics’ degradation. Recently, Han et al. (2020) found that a *Streptomyces* strain, isolated from agricultural soils, was able to grow with mulching film as carbon source and to thereby degrade the mulching film over time Therefore, we posit that *Streptomyces* is to some extent associated with PBAT’s degradation in the DPBOS treatment soil.

Further research work should focus on screening for mulching film-degrading fungi and bacteria. These candidates could then be inoculated back into soil to test their ability to improve degradation efficiency of the PBAT mulching film. Finally the mulching film-degrading fungi and bacteria could be prepared into product and put into soil to accelerate the degradation of mulching film. Our study provides a timely basis for the wider application of degradable PBAT mulching film towards the sustainable development of cotton crops.

In conclusion, application of different types of mulching films had different impact on soil microbial diversity and community. This study investigated the influence of traditional PE mulching film and degradable PBAT mulching film on cotton (*Gossypium* spp.) soil microbial diversity and community in Xinjiang Province, China. The PBAT mulching film could significantly alter the diversity of non-rhizosphere soil fungi when compared to using the PE mulching film. However, neither the PE nor PBAT mulching film had any significant influence on the diversity of soil bacteria and rhizosphere soil fungi. This study has provided an empirical basis for the further application of degradable PBAT mulching film for the sustainable development of cotton crops.

## Data Availability

All the sequences data were uploaded in NCBI Sequence Read Archive database under the BioProject numbers of PRJNA789993.
